# Lack of association between the chemokine receptor 5 polymorphism CCR5delta32 in rheumatoid arthritis and juvenile idiopathic arthritis

**DOI:** 10.1186/1471-2350-8-33

**Published:** 2007-06-12

**Authors:** Ewald Lindner, Gry BN Nordang, Espen Melum, Berit Flatø, Anne Marit Selvaag, Erik Thorsby, Tore K Kvien, Øystein T Førre, Benedicte A Lie

**Affiliations:** 1 Institute of Immunology, Rikshospitalet-Radiumhospitalet Medical Center, 0027 Oslo, Norway; 2 Department of Rheumatology, Rikshospitalet-Radiumhospitalet Medical Center, 0027 Oslo, Norway; 3 Department of Rheumatology, Diakonhjemmet Hospital, N-0319 Oslo, Norway

## Abstract

**Background:**

The chemokine receptor CCR5 has been detected at elevated levels on synovial T cells, and a 32 bp deletion in the *CCR5 *gene leads to a non-functional receptor. A negative association between the *CCR5Δ32 *and rheumatoid arthritis (RA) has been reported, although with conflicting results. In juvenile idiopathic arthritis (JIA), an association with CCR5 was recently reported. The purpose of this study was to investigate if the *CCR5Δ32 *polymorphism is associated with RA or JIA in Norwegian cohorts.

**Methods:**

853 RA patients, 524 JIA patients and 658 controls were genotyped for the *CCR5Δ32 *polymorphism.

**Results:**

The *CCR5Δ32 *allele frequency was 11.5% in the controls vs. 10.4% in RA patients (OR = 0.90; *P *= 0.36) and 9.7% in JIA patients (OR = 0.85; *P *= 0.20). No decreased homozygosity was observed for *CCR5Δ32*, as previously suggested.

**Conclusion:**

Our data do not support an association between the *CCR5Δ32 *allele and Norwegian RA or JIA patients. Combining our results with those from a recently published meta-analysis still provide evidence for a role for *CCR5Δ32 *in RA, albeit substantially weaker than the effect first reported.

## Background

In rheumatoid arthritis (RA) and juvenile idiopatic arthritis (JIA), the synovium is infiltrated with T cells and macrophages, and chemokine receptors and their ligands are involved in regulation of the inflammation [1,2]. Chemokines regulate lymphocyte trafficking by inducing cell motility and activating adhesion molecules. The chemokine receptor CCR5 is a G-protein-coupled seven-transmembrane-receptor, and it is expressed on monocytes, macrophages, memory T-cells and dendritic cells, where it serves as a receptor for RANTES, MIP-1-α, MIP-1-β and MCP [3]. CCR5 has functionally been implicated in the pathogenesis of RA [2] and JIA [4]. The expression of CCR5 is increased on synovial T cells in patients with JIA [4] or RA [5].

The gene encoding CCR5 is located on chomosome 3p21.3 [6]. A 32 base pair deletion (*CCR5Δ32*) in the *CCR5 *gene leads to a frame shift and a non-functional receptor. Homozygosity for *CCR5Δ32 *results in a complete lack of surface expression of CCR5 [7]. CCR5 happens to be a co-receptor for HIV-1 on CD4+ T-cells with the consequence that *CCR5Δ32 *homozygotes are protected against infections with HIV-1, while heterozygotes have a delay in the progression towards AIDS [8]. The *CCR5 *deletion is common among Caucasians, especially in Nordic populations [9], but has not been found in native Africans, American Indians or East Indian ethnic groups.

The role of *CCR5Δ32 *has been investigated genetically in several immune-mediated diseases. In RA, a negative association of the deletion has been reported with divergent results [10-14], while in childhood arthritis a weak negative association has been observed in a single study [15]. Regarding clinical outcome, it has been reported that carriers of the *CCR5Δ32 *allele occurred at a significantly higher frequency among patients with non-severe compared to severe disease (N = 160) [14], whereas a later and larger study of 438 patients did not find any association between carriage of the *CCR5Δ32 *and milder disease severity in inflammatory polyarthritis [16]. A recent meta-analysis of five reports addressing the association of *CCR5Δ32 *with susceptibility to develop RA in patients of European ancestry (1790 cases and 2717 controls), reported a significant negative association and estimated the deletion to provide disease resistance with an OR = 0.65 (95% CI [0.55–0.77])[17].

The aim of our study was to investigate if there is an association between *CCR5Δ32 *and RA in the largest individual data set studied so far, as well as to address whether *CCR5Δ32 *is also associated with JIA, not yet extensively investigated.

## Methods

853 RA patients, 524 JIA patients and 658 controls were included in the study. The RA patients, admitted to the Department of Rheumatology, Diakonhjemmet Hospital, Oslo, Norway, fulfilled the American College of Rheumatology (ACR) criteria [18] and the JIA patients, admitted to the Department of Rheumatology, Rikshospitalet-Radiumhospitalet Medical Center, Oslo, Norway, were classified according to the ILAR (International League of Associations for Rheumatology) criteria [19]. The control group was recruited through the Norwegian Bone Marrow Registry. All patients and controls were of Norwegian origin. The study was approved by the Norwegian Regional Ethics committee, and the participants had given their informed consent.

Whole-genome amplification was performed on genomic DNA using the Genomiphi^® ^kit (GE healthcare systems, Chalfont St. Giles, UK). Amplification of fragment surrounding the deletion was performed with the dye labelled forward primer 5'-ATCACTTGGGTGGTGGCTGTGTTTGCGTCTC and the reverse primer 5'-AGTAGCAGATGACCATGACAAGCAGCGGCAG under the following conditions; denaturation at 94°C for 15 seconds, annealing and extension at 70°C for 15 seconds, 30 cycles and final extension at 70°C for 5 min at a Perkin Elmer 9700 thermal cycler (Applied Biosystems, Foster City, CA, USA). Fragment length was determined on an ABI3730 DNA Analyzer (Applied Biosystems) with subsequent allele calling in GeneMapper v3.7 where a 193 bp PCR product corresponded to the wild type allele and a 161 bp product represented the deletion. Part of the control material was genotyped in our previous publication [20].

Statistical power was calculated prior to the study using the power calculator at [21], assuming the frequency of 10% for the *CCR5Δ32 *and an Odds Ratio of 0.56 (obtained from Zapico et al [14]). The power (at α = 0.05) was calculated to be 84% for the RA and 75% for the JIA data set. Allele and genotype frequencies in patients and controls were compared by the Chi-Square test, or Fischer's exact test when appropriate, using the Public Domain Software for Epidemiology and Disease Surveillance EPI Info Version 5 (Center of Disease Control, Epidemiology Program Office, Atlanta, GA, USA).

## Results and Discussion

The genotype and allele frequencies of *CCR5Δ32 *are summarised in Table 1. The genotyping success rate was >95% for both the patients and control populations, and all groups were in Hardy-Weinberg equilibrium. No significant negative association was observed with the Δ32 allele in either RA or JIA (Table 1), and only a negligible deviation in frequency was seen between the patients and controls. Furthermore, no decreased frequency for homozygosity of Δ32 was evident among the patients (0.9% in RA, 0.6% in JIA and 1.0% in controls). A revised meta-analysis, calculated by adding our results to those from the recently published meta-analysis of pooled RA cases and controls [17], reduced the initial published risk estimate from OR = 0.65; 95% CI [0.55–0.77], *p *< 0.0001, to OR = 0.83; 95% CI [0.73–0.95], *p = 0.0068*.

**Table 1:** *CCR5 *genotype and allele frequencies among controls, RA patients and JIA patients

	**Controls no. (%)**	**RA no. (%)**	**OR (95% CI)**	***p***	**JIA no. (%)**	**OR (95% CI)**	***p***
**CCR5 genotype**							
**Δ32/Δ32**	6 (0.9)	9 (1.0)	1.13 (0.43–2.98)	0.81	3 (0.6)	0.67 (0.20–2.31)	0.53
**Δ32/wt**	136 (21.1)	156 (18.7)	0.86 (0.66–1.11)	0.24	93 (18.1)	0.83 (0.62–1.11)	0.20
**wt/wt**	503 (78.0)	671 (80.3)	1.15 (0.89–1.48)	0.28	419 (81.4)	1.23 (0.92–1.64)	0.16
**CCR5 allele**							
**Δ32**	148 (11.5)	174 (10.4)	0.90 (0.71–1.13)	0.35	99 (9.7)	0.82 (0.63–1.07)	0.15
**wt**	1142 (88.5)	1498 (89.6)			931 (90.4)		

RA: rheumatoid arthritis; JIA: juvenile idiopathic arthritis; N: number of individuals/chromosomes with the denoted genotype/allele; OR (95% CI): Odds ratio with corresponding 95% confidence interval; wt: wild type; Δ32: 32 bp deletion.

Our results, from the largest cohort of RA patients and controls investigated to date, do not support the existence of a negative association between *CCR5Δ32 *and disease (OR = 0.90; 95% CI [0.71–1.14]). Neither was the proposed disease resistance provided by homozygosity of the deletion sustained in our data set. The frequency of *CCR5Δ32 *in our control population is in the same range as that observed in other European populations and is highly similar to that reported in a Danish population [11]. *CCR5Δ32 *has been found to have its highest prevalence among Nordic populations [9], which from a statistical power perspective should favour detection of a negative association in our Norwegian cohort, on the other hand deviating results between populations for disease associated alleles is not uncommon, e.g. for *PADI4 *[22-25].

Altogether, two studies suggest an association between *CCR5Δ32 *and RA [13,14], while three studies [10-12], in addition to ours, do not reveal statistical significant evidence to support this. The number of patients included in the positive studies was 160 and 516 whereas in the negative studies 278, 163, 673 and 853 patients were analysed. A weak effect of *CCR5Δ32 *on the disease risk, combined with inadequate power, could explain the contradictory results. Alternatively, there could be true differences between the populations in the effect exercised by *CCR5*; i.e. due to genetic or allelic heterogeneity, variable influence of environmental factors, gene-gene interaction, or variable linkage disequilibrium patterns. All studies display a deviation in the direction of a lower frequency for the deletion among RA patients, but in our data set only a 0.9% reduction in frequency was seen in RA patients compared to controls. The results of a recent meta-analysis of pooled cases and controls were in favour of a negative association (OR = 0.65 (95% CI [0.55–0.77])). [17]. We recalculated the power of our RA study post hoc based on the odds ratio for the *CCR5Δ32 *obtained in the recent meta-analysis [17], and given this risk effect our power was reduced to 63%. When our RA data set was included in a revised meta-analysis, the overall risk estimate was greatly reduced (OR = 0.83; 95% CI [0.73–0.95, *p *= 0.0068]). Furthermore, if the true risk estimate is in the range of that calculated after also including our data into the pooled meta-analysis, one would need almost 4000 cases and an equal amount of controls to replicate the finding with an 80% power. Notably, the *CCR5Δ32 *meta-analysis may also have been prone to type 1 error caused by e.g. publication bias or heterogeneity between the populations included in the combined analysis [26].

Our data does not reveal an association in JIA. After the initiation of our study, the possible association of *CCR5 *in JIA was addressed, and the authors reported a negative association between *CCR5Δ32 *and childhood arthritis, particularly in the early onset group and in those diagnosed with pauciarticular subtype [15]. This finding was not sustained in our data set as no association was revealed when stratifying for either JIA subgroups, including the oligoarthirits patients (comparable to the pauciarticular group defined by the ACR criterias; OR = 0.80, 95% CI [0.54–1.17], *p *= 0.23), or early disease onset (<6 years; OR = 0.81, 95% CI [0.55–1.19], *p *= 0.27). Further investigations are warranted for JIA before any firm conclusions can be drawn. Nevertheless, our study does not suggest that *CCR5Δ32 *influences the risk of JIA, and if present the effect is likely to be small.

CCR5 has functionally been implicated in RA, and our study does not formally exclude *CCR5 *as a candidate gene for RA and JIA. Polymorphisms located elsewhere in the gene could potentially be predisposing, and if the associations observed for Δ32 is only secondary due to linkage disequilibrium, that could be an explanation for the discrepant results obtained by different studies. Interestingly, in the recently reported childhood arthritis study [15] other polymorphisms in the *CCR5 *gene were also investigated, and an additional association with the CCR5 -1835T allele was also observed. No strong linkage disequilibrium between *CCR5Δ32 *and -1835T was evident, but the haplotype lacking both these alleles displayed positive association, which could suggest that the true etiological variant of *CCR5 *in RA or JIA pathogenesis that is still unidentified.

## Conclusion

In conclusion, whether the *CCR5 *gene is involved in RA or JIA susceptibility remains unclear, however, our study suggests that if a protective effect is exercised by CCR5*Δ32*, it is minor and less than predicted from the meta-analysis of previous publications[17].

## Competing interests

The author(s) declare that they have no competing interests.

## Authors' contributions

EL and GBNN participated in the design of the study, performed the genotyping, statistical analysis and drafted the manuscript. EM participated in the design of the study and performed the genotyping of a part of the control material. BF, AMS and ØTF contributed with DNA samples and clinical information for the JIA patients. TKK contributed with DNA samples and clinical information for the RA patients. ET helped writing the manuscript. BAL participated in the design of the study, statistical analysis and drafted the manuscript. All authors have read and approved the final manuscript.

## Pre-publication history

The pre-publication history for this paper can be accessed here:



## References

[B1] Pharoah DS, Varsani H, Tatham RW, Newton KR, de JW, Prakken BJ, Klein N, Wedderburn LR (2006). Expression of the inflammatory chemokines CCL5, CCL3 and CXCL10 in juvenile idiopathic arthritis, and demonstration of CCL5 production by an atypical subset of CD8+ T cells. Arthritis Res Ther.

[B2] Szekanecz Z, Kim J, Koch AE (2003). Chemokines and chemokine receptors in rheumatoid arthritis. Semin Immunol.

[B3] Wu L, LaRosa G, Kassam N, Gordon CJ, Heath H, Ruffing N, Chen H, Humblias J, Samson M, Parmentier M, Moore JP, Mackay CR (1997). Interaction of chemokine receptor CCR5 with its ligands: multiple domains for HIV-1 gp120 binding and a single domain for chemokine binding. J Exp Med.

[B4] Wedderburn LR, Robinson N, Patel A, Varsani H, Woo P (2000). Selective recruitment of polarized T cells expressing CCR5 and CXCR3 to the inflamed joints of children with juvenile idiopathic arthritis. Arthritis Rheum.

[B5] Suzuki N, Nakajima A, Yoshino S, Matsushima K, Yagita H, Okumura K (1999). Selective accumulation of CCR5+ T lymphocytes into inflamed joints of rheumatoid arthritis. Int Immunol.

[B6] Samson M, Soularue P, Vassart G, Parmentier M (1996). The genes encoding the human CC-chemokine receptors CC-CKR1 to CC-CKR5 (CMKBR1-CMKBR5) are clustered in the p21.3-p24 region of chromosome 3. Genomics.

[B7] Venkatesan S, Petrovic A, Van Ryk DI, Locati M, Weissman D, Murphy PM (2002). Reduced cell surface expression of CCR5 in CCR5Delta 32 heterozygotes is mediated by gene dosage, rather than by receptor sequestration. J Biol Chem.

[B8] Benkirane M, Jin DY, Chun RF, Koup RA, Jeang KT (1997). Mechanism of transdominant inhibition of CCR5-mediated HIV-1 infection by ccr5delta32. J Biol Chem.

[B9] Lucotte G (2001). Distribution of the CCR5 gene 32-basepair deletion in West Europe. A hypothesis about the possible dispersion of the mutation by the Vikings in historical times. Hum Immunol.

[B10] Cooke SP, Forrest G, Venables PJ, Hajeer A (1998). The delta32 deletion of CCR5 receptor in rheumatoid arthritis. Arthritis Rheum.

[B11] Garred P, Madsen HO, Petersen J, Marquart H, Hansen TM, Freiesleben SS, Volck B, Svejgaard A, Andersen V (1998). CC chemokine receptor 5 polymorphism in rheumatoid arthritis. J Rheumatol.

[B12] Gomez-Reino JJ, Pablos JL, Carreira PE, Santiago B, Serrano L, Vicario JL, Balsa A, Figueroa M, de J (1999). Association of rheumatoid arthritis with a functional chemokine receptor, CCR5. Arthritis Rheum.

[B13] Pokorny V, McQueen F, Yeoman S, Merriman M, Merriman A, Harrison A, Highton J, McLean L (2005). Evidence for negative association of the chemokine receptor CCR5 d32 polymorphism with rheumatoid arthritis. Ann Rheum Dis.

[B14] Zapico I, Coto E, Rodriguez A, Alvarez C, Torre JC, Alvarez V (2000). CCR5 (chemokine receptor-5) DNA-polymorphism influences the severity of rheumatoid arthritis. Genes Immun.

[B15] Prahalad S, Bohnsack JF, Jorde LB, Whiting A, Clifford B, Dunn D, Weiss R, Moroldo M, Thompson SD, Glass DN, Bamshad MJ (2006). Association of two functional polymorphisms in the CCR5 gene with juvenile rheumatoid arthritis. Genes Immun.

[B16] John S, Smith S, Morrison JF, Symmons D, Worthington J, Silman A, Barton A (2003). Genetic variation in CCR5 does not predict clinical outcome in inflammatory arthritis. Arthritis Rheum.

[B17] Prahalad S (2006). Negative association between the chemokine receptor CCR5-Delta32 polymorphism and rheumatoid arthritis: a meta-analysis. Genes Immun.

[B18] Arnett FC, Edworthy SM, Bloch DA, McShane DJ, Fries JF, Cooper NS, Healey LA, Kaplan SR, Liang MH, Luthra HS, . (1988). The American Rheumatism Association 1987 revised criteria for the classification of rheumatoid arthritis. Arthritis Rheum.

[B19] Petty RE, Southwood TR, Manners P, Baum J, Glass DN, Goldenberg J, He X, Maldonado-Cocco J, Orozco-Alcala J, Prieur AM, Suarez-Almazor ME, Woo P (2004). International League of Associations for Rheumatology classification of juvenile idiopathic arthritis: second revision, Edmonton, 2001. J Rheumatol.

[B20] Melum E, Karlsen TH, Broome U, Thorsby E, Schrumpf E, Boberg KM, Lie BA (2006). The 32-base pair deletion of the chemokine receptor 5 gene (CCR5-Delta32) is not associated with primary sclerosing cholangitis in 363 Scandinavian patients. Tissue Antigens.

[B21] Calculator P (2007). http://calculators.stat.ucla.edu/powercalc/.

[B22] Barton A, Bowes J, Eyre S, Spreckley K, Hinks A, John S, Worthington J (2004). A functional haplotype of the PADI4 gene associated with rheumatoid arthritis in a Japanese population is not associated in a United Kingdom population. Arthritis Rheum.

[B23] Martinez A, Valdivia A, Pascual-Salcedo D, Lamas JR, Fernandez-Arquero M, Balsa A, Fernandez-Gutierrez B, de la Concha EG, Urcelay E (2005). PADI4 polymorphisms are not associated with rheumatoid arthritis in the Spanish population. Rheumatology (Oxford).

[B24] Plenge RM, Padyukov L, Remmers EF, Purcell S, Lee AT, Karlson EW, Wolfe F, Kastner DL, Alfredsson L, Altshuler D, Gregersen PK, Klareskog L, Rioux JD (2005). Replication of putative candidate-gene associations with rheumatoid arthritis in >4,000 samples from North America and Sweden: association of susceptibility with PTPN22, CTLA4, and PADI4. Am J Hum Genet.

[B25] Suzuki A, Yamada R, Chang X, Tokuhiro S, Sawada T, Suzuki M, Nagasaki M, Nakayama-Hamada M, Kawaida R, Ono M, Ohtsuki M, Furukawa H, Yoshino S, Yukioka M, Tohma S, Matsubara T, Wakitani S, Teshima R, Nishioka Y, Sekine A, Iida A, Takahashi A, Tsunoda T, Nakamura Y, Yamamoto K (2003). Functional haplotypes of PADI4, encoding citrullinating enzyme peptidylarginine deiminase 4, are associated with rheumatoid arthritis. Nat Genet.

[B26] Salanti G, Sanderson S, Higgins JP (2005). Obstacles and opportunities in meta-analysis of genetic association studies. Genet Med.

